# Racial/Ethnic Health Disparities Among Rural Adults — United States, 2012–2015

**DOI:** 10.15585/mmwr.ss6623a1

**Published:** 2017-11-17

**Authors:** Cara V. James, Ramal Moonesinghe, Shondelle M. Wilson-Frederick, Jeffrey E. Hall, Ana Penman-Aguilar, Karen Bouye

**Affiliations:** 1 Centers for Medicare and Medicaid Services, Baltimore, Maryland; 2 Office of the Director, CDC, Atlanta, Georgia

## Abstract

**Problem/Condition:**

Rural communities often have worse health outcomes, have less access to care, and are less diverse than urban communities. Much of the research on rural health disparities examines disparities between rural and urban communities, with fewer studies on disparities within rural communities. This report provides an overview of racial/ethnic health disparities for selected indicators in rural areas of the United States.

**Reporting Period:**

2012–2015.

**Description of System:**

Self-reported data from the 2012–2015 Behavioral Risk Factor Surveillance System were pooled to evaluate racial/ethnic disparities in health, access to care, and health-related behaviors among rural residents in all 50 states and the District of Columbia. Using the National Center for Health Statistics 2013 Urban-Rural Classification Scheme for Counties to assess rurality, this analysis focused on adults living in noncore (rural) counties.

**Results:**

Racial/ethnic minorities who lived in rural areas were younger (more often in the youngest age group) than non-Hispanic whites. Except for Asians and Native Hawaiians and other Pacific Islanders (combined in the analysis), more racial/ethnic minorities (compared with non-Hispanic whites) reported their health as fair or poor, that they had obesity, and that they were unable to see a physician in the past 12 months because of cost. All racial/ethnic minority populations were less likely than non-Hispanic whites to report having a personal health care provider. Non-Hispanic whites had the highest estimated prevalence of binge drinking in the past 30 days.

**Interpretation:**

Although persons in rural communities often have worse health outcomes and less access to health care than those in urban communities, rural racial/ethnic minority populations have substantial health, access to care, and lifestyle challenges that can be overlooked when considering aggregated population data. This study revealed difficulties among non-Hispanic whites as well, primarily related to health-related risk behaviors. Across each population, the challenges vary.

**Public Health Action:**

Stratifying data by different demographics, using community health needs assessments, and adopting and implementing the National Culturally and Linguistically Appropriate Services Standards can help rural communities identify disparities and develop effective initiatives to eliminate them, which aligns with a *Healthy People 2020* overarching goal: achieving health equity.

## Introduction

Rural communities often are less racially and ethnically diverse than urban areas (*1*), have worse health outcomes (*2*,*3*), and have less access to care (*4*). Although less heterogeneous than urban areas, the demographic composition of rural communities is becoming more diverse, driven in large part by populations that have not historically lived in rural communities (*1*,*5*).

Racial/ethnic disparities in health and in quality of and access to health care are a well-documented and persistent problem (*6*,*7*). Across many indicators of health, access to care, and health care quality, racial/ethnic minorities fare worse than whites, and each population faces specific challenges (e.g., heart disease and stroke among blacks, tuberculosis among Asians, suicide among American Indians/Alaska Natives [AI/ANs], and having a regular source of care among Hispanics). Although health outcomes and quality of and access to care have improved for all populations, many racial/ethnic disparities remain (*6*,*7*). Despite the evidence of these disparities, few studies have examined racial/ethnic disparities within rural communities. Among those that have, many have focused their analyses on a particular state or geographic region and have been limited to one or two racial/ethnic populations (*8*).

The health and economic challenges faced by many rural residents in the United States have recently become the focus of growing national interest, creating an important opportunity to address many of these long-standing issues (*9*). To examine racial/ethnic health disparities in rural areas, the Centers for Medicare and Medicaid Services (CMS) and CDC analyzed 2012–2015 data from the Behavioral Risk Surveillance System (BRFSS) for all 50 states and the District of Columbia (DC). Racial/ethnic disparities across various indicators are characterized, including health-related quality of life, health care access and use, health-related behaviors, and chronic health conditions. The findings in this report can help public health officials, health providers, and other stakeholders develop solutions that achieve health equity, eliminate racial/ethnic disparities, and improve the health of all populations, which is an overarching goal of *Healthy People 2020* (*10*).

## Methods

BRFSS is an annual state-based, random-digit–dialed telephone (landline and cell telephone) survey of the noninstitutionalized U.S. population aged ≥18 years. CDC analyzed 2012–2015 self-reported BRFSS data from all 50 states and DC on demographic and health characteristics of adults in rural areas to evaluate racial/ethnic disparities in health, access to care, and health-related behaviors among rural residents. BRFSS data and documentation information, including survey questions, are available online (*11*). Response rates for BRFSS are calculated using standards set by the American Association of Public Opinion Research response rate formula #4 (*12*). The response rate is the number of respondents who completed the survey as a proportion of all eligible and likely eligible persons. The median weighted survey response rate for all states and DC in 2012–2015 ranged from 45.2% to 47.2%. For detailed information, see the BRFSS Summary Data Quality Reports for 2012–2015 (*11*). BRFSS data are weighted to represent state populations. In this study, data were combined for the 50 states and DC. A data set that identified counties in the 2012–2015 BRFSS was obtained from CDC through a data use agreement and was merged with BRFSS data. Urban-rural classification for county of residence was based on the 2013 NCHS Urban-Rural Classification Scheme for Counties. This classification scheme categorizes counties as large central metropolitan, large fringe metropolitan, medium metropolitan, small metropolitan, micropolitan, or noncore (rural). Of the 3,143 U.S. counties identified in 2013, a total of 1,325 were noncore counties, and 6.1% of the U.S. population lived in these counties (*13*). The study population included persons aged ≥18 years living in noncore counties in the 50 states and DC.

Demographic measures included race/ethnicity; age (18–44, 45–64, and ≥65 years), sex, educational attainment (less than high school, high school diploma or General Education Development [GED] certificate, some college, or college graduate), marital status (married or not married), household income (<$25,000, $25,000-$49,999, $50,000–$74,999, or ≥$75,000), employment status (employed or not employed), U.S. Census division (New England, Middle Atlantic, East North Central, West North Central, South Atlantic, East South Central, West South Central, Mountain, or Pacific), and U.S. census region (Northeast, Midwest, South, or West) (*14*). Categories for race/ethnicity included non-Hispanic white; non-Hispanic black; a combined Asian and Native Hawaiian/other Pacific Islander (NHOPI) category (used because of small numbers of respondents in the separate categories) that was also non-Hispanic; non-Hispanic AI/AN; and Hispanic or Latino (referred to as Hispanic in this report).

Health-related quality of life indicators included fair or poor health status, frequent mental distress (≥14 days in poor mental health in past 30 days), frequent physical distress (≥14 days in poor physical health in past 30 days), and activity limitations because of physical, mental, or emotional problems. (Respondents were asked whether they were limited in any way in any activities because of physical, mental, or emotional problems.) Health care access and use indicators included needing to see a physician in the past 12 months but being unable to do so because of cost, having any form of health care coverage, having at least one personal physician or other health care provider, how recently a physician was visited for a routine checkup (within past 2 years, at least 2 years ago, or never), and three cancer screening variables. Cancer screening included receiving a mammogram within the past 2 years among women aged 50–74 years; a Papanicolaou (Pap) test within the past 3 years among women aged 21–65 years; and colorectal cancer screening among adults aged 50–75 years (received at least one colorectal cancer screening test recommended by the U.S. Preventive Services Task Force within the recommended time interval: colonoscopy within the past 10 years, fecal occult blood test within the past year, or sigmoidoscopy within the past 5 years in combination with fecal occult blood test within the past 3 years).

Health-related behaviors included in the study were cigarette smoking, binge drinking, and lack of leisure-time physical activity. Respondents were classified as current smokers (≥100 cigarettes in lifetime), former smokers (≥100 cigarettes in lifetime but quit), and never smokers. Binge drinking was defined as five or more drinks (men) or four or more drinks (women) on any occasion in the past 30 days. No leisure-time physical activity was defined as not participating in any physical activity or exercise outside of the respondent’s regular job in the past month.

For chronic health conditions, the number of conditions from a list of 12 was totaled and categorized as none, one, or two or more. These conditions included myocardial infarction; coronary heart disease; stroke; hypertension; asthma; skin cancer; other types of cancer; chronic obstructive pulmonary disease; depressive disorder; kidney disease; diabetes; and some form of arthritis, rheumatoid arthritis, gout, lupus, or fibromyalgia. (Arthritis diagnoses included diagnoses such as rheumatism; polymyalgia rheumatica; osteoarthritis [not osteoporosis]; tendonitis, bursitis, bunions, and tennis elbow; carpal tunnel syndrome and tarsal tunnel syndrome; and joint infection.)

 Depending on the condition, respondents were asked whether they had ever received a diagnosis of or been told by a physician, a nurse, or another health professional that they had the condition. Respondents who indicated either yes or no for each of the 12 chronic conditions were included; those who answered “don’t know” or declined to answer any of the items were excluded. Depression, which was one of the 12 conditions, also was analyzed separately. The study also included obesity (not one of the 12 conditions), defined as body mass index (BMI) ≥30 kg/m^2^; BMI ≥40 kg/m^2^ was categorized as severe obesity.

Cancer screening rates were estimated using 2012 and 2014 BRFSS data because screening variables were not available for most states for other years. The number of chronic conditions was estimated using BRFSS data from 2013 and 2015 only to allow inclusion of hypertension, an important chronic condition on which data are only collected in the rotating core of BRFSS during odd calendar years. Prevalence of respondent characteristics was estimated with 95% confidence intervals (CIs). Statistical significance (p<0.05) of differences in estimated prevalence (comparing other race/ethnicity groups to non-Hispanic whites in a pairwise manner) was assessed by t-tests. Estimates with a relative standard error >30% were considered unstable and were suppressed. Non-Hispanic white was selected as the reference group not as a benchmark for good health but because it represented the largest proportion of the population. Estimates were age adjusted to the U.S. 2000 population aged ≥18 years with the direct method (*15*), except for demographic and screening variables. Samples weights for study variables were adjusted for pooling multiple years of BRFSS survey data. SAS-callable SUDAAN, version 11.0.1 (Research Triangle Institute, Research Triangle Park, NC), which takes into account the complex sampling design of BRFSS, was used for the analyses.

## Results

The study sample for most variables contained 263,054 adult respondents in rural counties in the 50 states and DC. The sample included the following: 231,221 non-Hispanic white (87.90%), 12,751 non-Hispanic black (4.85%), 10,947 AI/AN (4.16%), 7,223 Hispanic (2.75%), and 912 Asian and NHOPI (0.35%).

### Demographics

Compared with rural non-Hispanic whites, rural racial/ethnic minorities tended to be younger, to have less income, and to have lower levels of educational attainment (Table 1). More non-Hispanic blacks (43.7%), Hispanics (66.0%), Asians and NHOPIs (60.5%), and AI/ANs (49.3%) were aged 18–44 years than non-Hispanic whites (36.9%). Fewer Hispanics (6.2%), non-Hispanic blacks (8.4%), AI/ANs (8.5%) were college graduates, compared with non-Hispanic whites (16.0%). Conversely, more Asians and NHOPIs (35.4%) were college educated. More non-Hispanic blacks (61.8%), AI/ANs (56.3%), and Hispanics (53.1%) had annual household incomes <$25,000 than non-Hispanic whites (31.8%). Compared with non-Hispanic whites (52.3%), employment rates were lower for non-Hispanic blacks (46.3%) and AI/ANs (45.0%) but higher for Asians and NHOPIs (65.2%) and Hispanics (61.1%).

**TABLE 1:** Sociodemographic characteristics of adults aged ≥18 years living in rural areas, by race/ethnicity* — Behavioral Risk Factor Surveillance System,^†^ United States, 2012–2015

Characteristic	Black % (95% CI)	White % (95% CI)	Hispanic % (95% CI)	Asian or NHOPI % (95% CI)	AI/AN % (95% CI)	Total
**Age (yrs)**						
18–44	43.7^§^ (42.0–45.4)	36.9 (36.5–37.4)	66.0^§^ (63.9–68.1)	60.5^§^ (51.4–68.9)	49.3^§^ (46.9–51.6)	**39.7 (39.2–40.1)**
45–64	38.9 (37.4–40.5)	37.4(37.0,37.8)	25.4^§^ (23.5–27.3)	32.0 (23.5–41.9)	37.0 (34.8–39.3)	**36.7 (36.3–37.1)**
≥65	17.4^§^ (16.4–18.4)	25.7 (25.4–25.7)	8.6^§^ (7.6–9.7)	7.5^§^ (5.3–10.6)	13.7^§^ (12.4–15.1)	**23.6 (23.3–23.9)**
**Sex**						
Male	45.9^§^ (44.3–47.6)	48.4 (48.0–48.9)	54.7^§^ (52.4–57.0)	50.2 (41.8–58.5)	48.8 (46.4–51.2)	**48.6 (48.2–49.0)**
Female	54.1^§^ (52.4–55.7)	51.6 (51.1–52.0)	45.3^§^ (43.0–47.6)	49.8 (41.5–58.2)	51.2 (48.8–53.6)	**51.4 (50.9–51.8)**
**Marital status**						
Not married	68.1^§^ (66.6–69.5)	41.0 (40.5–41.4)	51.0^§^ (48.6–53.4)	43.8 (35.9–52.0)	61.1^§^ (58.9–63.4)	**44.3 (43.9–44.7)**
Married	31.9^§^ (30.5–33.4)	59.0 (58.6–59.5)	49.0^§^ (46.6–51.4)	56.2 (48.0–64.0)	38.8^§^ (36.6–41.1)	**55.7 (55.2–56.1)**
**Educational attainment**						
<High school	28.3^§^ (26.8–29.9)	15.3 (14.9–15.7)	44.2^§^ (41.8–46.6)	—^¶^	25.9 (23.7–28.3)^§^	**18.3 (18.0–18.7)**
High school diploma/GED	40.1^§^ (38.5–41.7)	37.7 (37.3–38.1)	31.4^§^ (29.3–33.7)	29.1^§^ (22.3–37.0)	35.9 (33.7–38.2)	**37.4 (37.0–37.8)**
Some college	23.1^§^ (21.8–24.5)	31.0 (30.6–31.4)	18.2^§^ (16.5–20.0)	22.7^§^ (17.3–29.2)	29.6 (27.5–31.9)	**29.5 (29.1–29.8)**
College graduate	8.4^§^ (7.8–9.2)	16.0 (15.8–16.3)	6.2^§^ (5.4–7.2)	35.4^§^ (28.2–43.3)	8.5^§^ (7.5–9.7)	**14.8 (14.5–15.0)**
**Annual household income**						
<$25,000	61.8^§^ (60.1–63.6)	31.8 (31.3–32.2)	53.1^§^ (50.5–55.7)	28.6 (21.6–36.7)	56.3^§^ (53.7–58.9)	**36.1 (35.7–36.5)**
$25,000–$49,999	25.2^§^ (23.7–26.8)	30.7 (30.3–31.1)	27.7^§^ (25.5–30.0)	25.6 (18.7–34.0)	25.1^§^ (23.0–27.4)	**29.9 (29.5–30.3)**
$50,000–$74,999	7.4^§^ (6.5–8.4)	16.8 (16.5–17.2)	10.6^§^ (9.0–12.5)	—	9.6^§^ (8.0–11.5)	**15.5 (15.2–15.8)**
≥$75,000	5.5^§^ (4.9–6.3)	20.7 (20.4–21.1)	8.6^§^ (7.2–10.2)	28.5 (20.6–38.0)	8.9^§^ (7.8–10.3)	**18.5 (18.2–18.9)**
**Employment status**						
Not employed	53.7^§^ (52.1–55.4)	47.7 (47.2–48.1)	38.9^§^ (36.6–41.2)	34.8^§^ (27.9–42.3)	55.0^§^ (52.6–57.4)	**47.8 (47.3–48.2)**
Employed	46.3^§^ (44.6–47.9)	52.3 (51.9–52.8)	61.1^§^ (58.8–63.4)	65.2^§^ (57.7–72.1)	45.0^§^ (42.6–47.4)	**52.2 (51.8–52.6)**
Census division**						
New England	0.2^§^ (0.1–0.3)	3.7 (3.6–3.8)	0.6^§^ (0.5–0.8)	2.3^§^ (1.6–3.2)	1.7^§^ (1.4–2.0)	**3.2 (3.1–3.2)**
Middle Atlantic	1.0^§^ (0.6–1.6)	5.6 (5.4–5.9)	2.5^§^ (1.7–3.7)	—	2.7^§^ (1.6–4.3)	**5.0 (4.8–5.2)**
East North Central	1.9^§^ (1.4–2.7)	17.8 (17.4–18.1)	5.6^§^ (4.5–6.9)	9.6^§^ (6.3–14.3)	8.1^§^ (6.6–9.9)	**15.4 (15.1–15.8)**
West North Central	2.1^§^ (1.7–2.5)	18.9 (18.6–19.1)	9.4^§^ (8.5–10.3)	15.9 (12.1–20.7)	16.9^§^ (15.7–18.1)	**16.8 (16.6–17.0)**
South Atlantic	45.4^§^ (43.8–47.0)	16.4 (16.1–16.7)	16.1 (14.4–18.0)	15.0 (10.5–20.9)	8.6 (7.1–10.3)^§^	**18.6 (18.2–18.9)**
East South Central	29.6^§^ (28.3–30.9)	14.9 (14.6–15.1)	4.4^§^ (3.6–5.4)	4.5^§^ (2.9–7.0)	5.4^§^ (4.4–6.6)	**15.2 (14.9–15.4)**
West South Central	18.9^§^ (17.6–20.3)	12.6 (12.3–12.9)	38.5^§^ (36.2–41.0)	17.5 (11.8–25.2)	18.7^§^ (16.9–20.6)	**14.9 (14.5–15.2)**
Mountain	0.3^§^ (0.2–0.50)	6.3 (6.2–6.4)	14.1^§^ (13.2–15.1)	6.9 (5.1–9.2)	24.4^§^ (22.5–26.5)	**6.7 (6.6–6.8)**
Pacific	0.7^§^ (0.3–1.4)	3.9 (3.7–4.1)	8.8^§^ (7.3–10.5)	21.8^§^ (13.6–33.0)	13.8^§^ (12.3–15.4)	**4.3 (4.1–4.5)**
**Census region**						
Northeast	1.2^§^ (0.8–1.8)	9.3 (9.1–9.6)	3.1^§^ (2.3–4.3)	8.8 (5.5–13.9)	4.3^§^ (3.2–5.8)	**8.1 (7.9–8.4)**
Midwest	4.0^§^ (3.3–4.8)	36.6 (36.3–37.0)	14.9^§^ (13.6–16.4)	25.5^§^ (20.1–31.8)	25.0^§^ (23.1–26.9)	**32.3 (31.9–32.6)**
South	93.9^§^ (92.8–94.8)	43.9 (43.5–44.3)	59.1^§^ (56.8–61.3)	37.0 (29.6–45.2)	32.5^§^ (30.3–34.9)	**48.6 (48.2–49.0)**
West	1.0^§^ (0.6–1.7)	10.2 (9.9–10.4)	22.9^§^ (21.2–24.7)	28.7^§^ (20.3–38.7)	38.2^§^ (36.0–40.5)	**11.0 (10.7–11.2)**

**Abbreviations:** AI/AN = American Indian/Alaska Native; CI = confidence interval; GED = General Education Development certificate; NHOPI = Native Hawaiian/other Pacific Islander.

* All races are non-Hispanic.

^†^ BRFSS data and documentation information, including survey questions, are available. (CDC. Behavioral Risk Factor Surveillance System: survey data & documentation. Atlanta, GA: CDC, US Department of Health and Human Services; 2017. https://www.cdc.gov/brfss/data_documentation/index.htm).

^§^ Significantly different (p<0.05, t-test) from non-Hispanic white.

^¶^ Estimates not reported because relative standard error was >30%.

** Census divisions are categorized into U.S. census regions: New England and Middle Atlantic (Northeast); East North Central and West North Central (Midwest); South Atlantic, East South Central, and West South Central (South); Mountain and Pacific (West) (**Source:** US Census Bureau. Census regions and divisions of the United States. Washington, DC: US Census Bureau. https://www2.census.gov/geo/pdfs/maps-data/maps/reference/us_regdiv.pdf).

Substantial variation was found by race/ethnicity in census region and division of residence. The majority of Hispanic blacks (93.9%) and approximately 60% of Hispanics (59.1%) lived in the southern region. Within this region, additional variations were found, with 45.4% of all non-Hispanic blacks in the study living in the South Atlantic division and 38.5% of all Hispanics living in the West South Central division. A total of 21.8% of Asians and NHOPIs lived in the Pacific division, and 37.0% lived in the southern region. Although 38.2% of AI/ANs lived in the western region; 24.4%, 18.7%, and 16.9% lived in the Mountain, West South Central, and West North Central divisions, respectively. Many non-Hispanic whites (43.9%) lived in the southern region; however, approximately one fifth lived in each of the following divisions: East North Central (17.8%) and West North Central (18.9%).

### Health-Related Quality of Life

Rates of self-reported fair or poor health were higher among AI/ANs (28.9%), non-Hispanic blacks (28.8%), and Hispanics (28.4%) and lower among Asians and NHOPIs (10.4%) compared with those for non-Hispanic whites (18.5%) (Table 2). Compared with non-Hispanic whites (12.5%), the prevalence of frequent mental distress was higher among AI/ANs (17.1%) and non-Hispanic blacks (13.9%) but lower among Asians and NHOPIs (5.4%).

**TABLE 2:** Health and health care characteristics of adults aged ≥18 years living in rural areas,* by race/ethnicity^†^ — Behavioral Risk Factor Surveillance System,^§^ United States, 2012–2015

Characteristic	Black % (95% CI)	White % (95% CI)	Hispanic % (95% CI)	Asian or NHOPI % (95% CI)	AI/AN % (95% CI)	Total
**Health-related quality of life**						
Health status: fair or poor	28.8^¶^ (27.4–30.2)	18.5 (18.1–18.9)	28.4^¶^ (26.4–30.4)	10.4^¶^ (7.1–15.0)	28.9^¶^ (26.8–31.1)	**20.1 (19.7–20.4)**
Frequent physical distress (≥14 days in poor physical health in past 30 days)	15.9^¶^ (14.8–17.0)	13.3 (12.9,13.6)	13.9 (12.4–15.6)	7.2^¶^ (4.3–11.6)	19.6^¶^ (17.8–21.5)	**13.5 (13.2–13.8)**
Frequent mental distress (≥14 days in poor mental health in past 30 days)	13.9^¶^ (12.7–15.1)	12.5 (12.2–12.9)	11.2 (9.9–12.7)	5.4^¶^ (3.3–8.6)	17.1^¶^ (15.3–19.1)	**12.5 (12.2–12.8)**
Activity limitation because of physical, mental, or emotional problems	23.4 (22.2–24.8)	22.3 (21.9–22.7)	15.4^¶^ (13.9–17.1)	13.8^¶^ (9.6–19.3)	28.5^¶^ (26.4–30.8)	**22.1 (21.7–22.4)**
**Health care access and use**						
Could not see doctor in past 12 months because of cost	24.5^¶^ (23.0–26.0)	15.0 (14.6–15.4)	23.1^¶^ (21.3–25.1)	17.2 (11.0–25.8)	19.1^¶^ (17.1–21.2)	**16.4 (16.1–16.8)**
Health care coverage	73.2^¶^ (71.5–74.8)	83.9 (83.5–84.3)	61.1^¶^ (58.9–63.2)	81.2 (75.5–85.9)	84.8 (82.7–86.7)	**81.2 (80.8–81.6)**
At least one personal doctor or health care provider	76.7^¶^ (75.0–78.3)	78.6 (78.1–79.1)	61.5^¶^ (59.3–63.6)	64.8^¶^ (55.8–72.9)	63.7^¶^ (61.4–66.0)	**76.6 (76.1–77.0)**
Length of time since last routine checkup						
Never	0.5^¶^ (0.3–0.8)	1.6 (1.4–1.7)	4.6^¶^ (3.8–5.5)	—**	1.9 (1.3–2.8)	**1.7 (1.6–1.9)**
≤2 yrs	87.5^¶^ (86.2–88.8)	77.6 (77.1–78.1)	72.9^¶^ (70.7–74.9)	72.6 (63.1–80.4)	76.8 (74.6–78.9)	**78.1 (77.7–78.5)**
>2 yrs	12.0^¶^ (10.8–13.3)	20.9 (20.4–21.3)	22.6 (20.7–24.7)	23.9 (16.3–33.6)	21.2 (19.2–23.4)	**20.2 (19.8–20.6)**
Cancer screening^††^						
Papanicolaou test during past 3 yrs, women aged 21–65 yrs	82.3 (78.4–85.6)	78.6 (77.6–79.6)	78.5 (73.9–82.6)	—	77.6 (72.5–82.0)	**78.6 (77.6–79.6)**
Mammogram during past 2 yrs, women aged 50–74 yrs	77.2^¶^ (74.0–80.1)	73.4 (72.5–74.3)	60.1^¶^ (52.1–67.6)	67.5 (50.4–80.9)	68.1 (61.2–74.3)	**73.1 (72.3–74.0)**
Met colorectal cancer screening recommendation, adults aged 50–75 yrs	53.6^¶^ (50.4–56.7)	61.7 (60.9–62.5)	43.4^¶^ (37.5–49.6)	53.4 (29.2–76.0)	53.4^¶^ (48.2–58.6)	**60.3 (59.5–61.0)**
**Chronic health conditions^§§^**						
Number of chronic conditions						
None	35.4 (33.0–37.8)	37.8 (37.1–38.5)	49.2^¶^ (46.3–52.2)	61.8^¶^ (52.9–70.0)	34.0^¶^ (30.8–37.4)	**38.6 (37.9–39.3)**
One	24.3 (22.2–26.5)	26.3 (25.6–26.9)	23.4^¶^ (20.9–26.1)	17.5^¶^ (12.3–24.2)	25.7 (22.5–29.2)	**25.7 (25.1–26.3)**
Two or more	40.3^¶^ (38.2–42.5)	36.0 (35.3–36.6)	27.4^¶^ (24.9–30.0)	20.7^¶^ (14.6–28.5)	40.3^¶^ (37.3–43.4)	**35.7 (35.1–36.2)**
Depressive disorder	15.8^¶^ (14.6–17.1)	20.3 (19.9–20.8)	15.9^¶^ (14.4–17.6)	5.8^¶^ (3.8–8.7)	23.2^¶^ (21.2–25.4)	**19.5 (19.1–19.9)**
Obesity						
BMI ≥30 kg/m^2^	45.9^¶^ (44.1–47.7)	32.0 (31.6–32.5)	35.5^¶^ (33.2–37.9)	15.5^¶^ (11.4–20.7)	38.5^¶^ (36.2–40.9)	**33.4 (33.0–33.9)**
BMI ≥40 kg/m^2^	12.1^¶^ (10.9–13.4)	5.0 (4.8–5.2)	5.1 (4.1–6.2)	—	6.9^¶^ (5.8–8.2)	**5.6 (5.4–5.8)**
**Health behaviors**						
Cigarette smoking						
Never	60.9^¶^ (59.1–62.6)	50.6 (50.1–51.1)	61.7^¶^ (59.5–64.0)	74.0^¶^ (68.0–79.3)	39.4^¶^ (37.1–41.8)	**52.2 (51.8–52.7)**
Former	15.9^¶^ (14.9–17.1)	24.7 (24.3–25.1)	21.3^¶^ (19.4–23.2)	15.1^¶^ (11.2–20.2)	23.9 (21.8–26.0)	**23.7 (23.3–24.1)**
Current	23.2 (21.7–24.8)	24.7 (24.2–25.2)	17.0^¶^ (15.4–18.7)	10.9^¶^ (7.5–15.4)	36.7^¶^ (34.4–39.2)	**24.1 (23.6–24.5)**
Binge drinking^¶¶^	11.7^¶^ (10.5–13.1)	16.3 (15.9–16.7)	14.3^¶^ (12.7–16.0)	9.9^¶^ (6.8–14.3)	15.6 (14.0–17.5)	**15.66 (15.3–16.0)**
No leisure-time physical activity in past month	38.2^¶^ (36.5–40.0)	27.7 (27.3–28.2)	35.4^¶^ (33.2–37.7)	27.6 (20.7–35.7)	29.8 (27.6–32.1)	**29.2 (28.8–29.7)**

**Abbreviations:** AI/AN = American Indian/Alaska Native; CI = confidence interval; NHOPI = native Hawaiian/other Pacific Islander.

*Percentages for adults aged ≥18 years are age-adjusted to the U.S. 2000 population aged ≥18 years with the direct method, with the exception of screening variables.

^†^ All races are non-Hispanic.

^§^ BRFSS data and documentation information, including survey questions, are available. (CDC. Behavioral Risk Factor Surveillance System: survey data & documentation. Atlanta, GA: CDC, US Department of Health and Human Services; 2017. https://www.cdc.gov/brfss/data_documentation/index.htm).

^¶^ Significantly different (p<0.05, t-test) from non-Hispanic white.

** Estimates not reported because relative standard error was >30%.

^††^ Screening variables rely on 2012 and 2014 data only. Adults who met colorectal cancer screening recommendations refer to adults aged 50–75 years who received at least one colorectal cancer screening test recommended by the U.S. Preventive Services Task Force within the recommended time interval (colonoscopy within the past 10 years, fecal occult blood test within the past year, or sigmoidoscopy within the past 5 years in combination with fecal occult blood test within the past 3 years).

^§§^ Chronic conditions considered include myocardial infarction; coronary heart disease; stroke; hypertension; asthma; skin cancer; other types of cancer; chronic obstructive pulmonary disease; depressive disorder; kidney disease; some form of arthritis, rheumatoid arthritis, gout, lupus, or fibromyalgia; and diabetes. Estimates were obtained using data from 2013 and 2015 because data on hypertension were not available for 2012 and 2014.

^¶¶ ^Binge drinking was defined as five or more drinks (men) or four or more drinks (women) on any occasion in the past 30 days.

### Health Care Access and Use

Fewer non-Hispanic blacks (73.2%) and Hispanics (61.1%) reported having health care coverage compared with non-Hispanic whites (83.9%). More non-Hispanic blacks (24.5%), Hispanics (23.1%), and AI/ANs (19.1%) said they could not see a physician when needed because of cost than non-Hispanic whites (15.0%). This was consistent with findings regarding having a personal health care provider (76.7%, 61.5%, and 63.7% for non-Hispanic blacks, Hispanics and AI/ANs, respectively, versus 78.6% for non-Hispanic whites); Asians and NHOPIs (64.8%) also less often had a personal health care provider, compared with non-Hispanic whites (even though they did not differ significantly from non-Hispanic whites with regard to health care coverage). More non-Hispanic black women (77.2%) but fewer Hispanic women (60.1%) had a mammogram in the past 2 years (among women aged 50–74 years) compared with non-Hispanic whites (73.4%). Fewer non-Hispanic blacks (53.6%), Hispanics (43.4%), and AI/ANs (53.4%) than non-Hispanic whites (61.7%) were up to date with colorectal cancer screening (among those aged 50–75 years).

### Health-Related Behaviors

Fewer Hispanics (17.0%) and Asians and NHOPIs (10.9%) were current smokers than non-Hispanic whites (24.7%). Similarly, the prevalence of binge drinking was lower among Hispanics (14.3%), non-Hispanic blacks (11.7%), and Asians and NHOPIs (9.9%) than among non-Hispanic whites (16.3%).

### Chronic Health Conditions

More non-Hispanic blacks and AI/ANs (40.3% of both groups) than non-Hispanic whites (36.0%) reported having multiple chronic health conditions, and fewer Hispanics (27.4%) and Asians and NHOPIs (20.7%) reported this. Depression was more common among AI/ANs (23.2%) than among non-Hispanic whites (20.3%) and less common among non-Hispanic blacks (15.8%), Hispanics (15.9%), and Asians and NHOPIs (5.8%). Obesity (35.5%) but not severe obesity (5.1%) was more prevalent among Hispanics than among non-Hispanic whites; obesity (15.5%) was less prevalent among Asians and NHOPIs. Both obesity and severe obesity were more prevalent among non-Hispanic blacks (45.9% and 12.1%) and AI/ANs (38.5% and 6.9%) compared with non-Hispanic whites (32.0% and 5.0%).

## Discussion

Researchers often refer to the differences between rural and urban communities when discussing disparities in rural health; less frequently discussed are the racial/ethnic disparities experienced within rural communities. The results of this study indicate that assessing rural data at only the population level prevents identification of important disparities. These results underscore that race/ethnicity should be considered when assessing differences within rural communities. The results also indicate that regardless of race/ethnicity, all rural populations experience health problems, and the nature of those problems differs. AI/ANs experienced more problems related to health-related quality of life and had higher rates of ever having depression or being a current smoker than non-Hispanic whites. Asians and NHOPIs experienced challenges in health care access. For example, compared with non-Hispanic whites, fewer Asians and NHOPIs reported having a personal health care provider. Non-Hispanic whites had higher rates of binge drinking than all groups except for AI/ANs, for which the comparison was not statistically significant. In this analysis, the only comparisons tested were between non-Hispanic whites and other racial/ethnic groups. However, to reflect the wide range of experiences, the estimated prevalence of obesity among non-Hispanic blacks was 45.9%; among other groups, this ranged from 15.5% among Asians and NHOPIs to 38.5% among AI/ANs. For severe obesity, the prevalence among non-Hispanic blacks was 12.1% and ranged from 5.0% among non-Hispanic whites to 6.9% among AI/ANs. The health coverage rate for Hispanics was 61.1%; coverage ranged from 73.2% among non-Hispanic blacks to 84.8% among AI/ANs. Whereas 4.6% of Hispanics had never received a routine check-up, the prevalence among other groups ranged from 0.5% among non-Hispanic blacks to 1.9% among AI/ANs.

Research specifically focused on racial/ethnic health disparities in rural areas is limited. The findings in this report are generally consistent with those described elsewhere. First, previous reports indicated that rural racial/ethnic minorities were more likely to be uninsured than whites (*16*) and that identifying as a minority was associated with reports of fair or poor health (*17*). In this report, compared with non-Hispanic whites, non-Hispanic blacks and Hispanics more often were uninsured, and all minorities except Asians and NHOPIs more often reported their health as fair or poor. Second, non-Hispanic blacks previously have been found to have the highest obesity prevalence among populations examined (*18*), which was consistent with this study. Third, similar to this report, a previous study of prevalence and trends in smoking by race/ethnicity found the prevalence of current smoking to be lowest among Asians and highest among AI/ANs (*19*).

Unhealthy behaviors and social circumstances contribute proportionately more to premature death than does inadequate health care (*20*). This study found significant racial/ethnic differences in the social circumstances of rural residents. Rural racial/ethnic minorities tended to be younger than non-Hispanic whites, and rural non-Hispanic blacks, Hispanics, and AI/ANs tended to be poorer and have lower educational attainment. The analysis also indicated that rural non-Hispanic blacks were clustered in the South (93.9%), with nearly half (45.4%) living in the South Atlantic census division. In contrast, the largest proportion of rural Hispanics lived in the West South Central division (38.5%), whereas the largest proportion of rural Asians and NHOPIs and rural AI/ANs lived in the Pacific and Mountain divisions, respectively. Disparities identified in this report might vary by geographic region; however, this was not analyzed. More research is needed to understand the interaction between race/ethnicity and geography (i.e., regional, state, and local influences). As programs and policies are being developed to improve health and access to care in rural communities, understanding and addressing the unique needs of each racial/ethnic population and the variations in the social determinants of health within and among groups are important for these programs and policies to be successful.

Like their urban counterparts, rural communities are becoming increasingly diverse. Moreover, the composition of the diversity by race/ethnicity is different from that previously documented for rural areas. Historically, non-Hispanic blacks have represented the largest minority population in rural communities; non-Hispanic blacks were the largest group in rural and small-town areas according to the 2000 U.S. census. However, by the 2010 census, Hispanics had become the largest group (*1*). Moreover, Hispanics, Asians, and NHOPIs represented more than half of the population growth in rural communities (*1*). Although rural Hispanics and Asians and NHOPIs in this report were similar in some ways (e.g., their age distribution and employment status), in others, they were very different. Whereas 35.4% and 28.5% of Asians and NHOPIs were estimated to have the most educational attainment and income, respectively, this was true for only 6.2% and 8.6% of Hispanics. Asians and NHOPIs also tended to have better health outcomes. Changing demographics can lead to unmet health needs among new and growing populations if health care providers do not work to understand and address the unique needs of the populations they encounter.

An overarching goal of *Healthy People 2020* is to achieve health equity and eliminate health disparities (*10*). To reach this goal and improve the health of a population, providers and other stakeholders need to know what the needs are and how they can work with community groups and others to address them.

Every 3 years, nonprofit hospitals are required to conduct a community health needs assessment and demonstrate that they engaged the community in the process (*21*). In this case, the community refers to state, local, tribal, or regional public health departments and medically underserved, low-income, and minority populations (*22*). The Association of State and Territorial Health Officials encourages collaboration between hospitals and public health departments to conduct the community health needs assessment (*23*).

When a community understands the health needs of its population, stakeholders can focus on developing an action plan to address them that includes a focus on organizational readiness and capacity to address health disparities. Stakeholders also can implement the National Culturally and Linguistically Appropriate Services Standards (*24*) to increase their ability to address the health needs of racially and ethnically diverse populations, thereby enhancing the likelihood that programs not only are effective for all populations served but also reduce disparities.

Organizations ready to take action to reduce disparities have numerous other resources available to them as well. The U.S. Department of Health and Human Services Action Plan to Reduce Racial and Ethnic Disparities (*25*) and the National Partnership for Action (*26*) identify specific actions for stakeholders. CMS encourages stakeholders to use resources from Building an Organizational Response to Health Disparities, such as the disparities action statement (*27*), to identify, prioritize, and develop a plan to address health disparities.

## Limitations

The findings in this report are subject to several limitations. First, BRFSS responses are self-reported and subject to reporting bias (including recall bias, which might affect the accuracy of reporting of past events and behaviors, and social desirability bias, which could result in underreporting behaviors such as smoking). Second, BRFSS only includes noninstitutionalized adults; therefore, results might not reflect the experiences of institutionalized persons. Third, relatively low state response rates might have affected the findings, potentially leading to overestimates or underestimates. However, numerous studies have shown national estimates to be reliable and valid (*28*). Fourth, cervical cancer screening recommendations changed in 2012 to include human papillomavirus (HPV) testing in combination with Pap testing; questions on HPV testing were not asked in all states until the 2016 BRFSS. Therefore, the prevalence estimates provided in this report might be slight underestimates. Fifth, because high blood cholesterol levels and certain other chronic conditions were not included, the percentages of respondents indicating they had been told they had one or two or more chronic conditions are likely to be underestimates. Sixth, although Office of Management and Budget data standards separate response categories for NHOPIs and Asians (*29*), these two groups were combined in this report because of small numbers of respondents. Although aggregating in this manner avoided these respondents being placed in an “other” category for race/ethnicity, doing this presumably obscured differences between the groups. Finally, the health and health care characteristics examined were not stratified by geographic area, and regional variation in health disparities was not examined in this report.

## Future Directions

Numerous racial/ethnic health disparities within rural areas of the United States were identified in this report, as were variations in how racial/ethnic populations are distributed across the United States. Health outcomes and social policies also vary across the United States, and additional research is needed to understand the complex relationship between geography and disparities. Future research could include conducting multivariate analyses to clarify the associations among race/ethnicity, geography, and health status or could focus on understanding the diversity among Asians and NHOPIs (groups that could not be disaggregated within this report) and within racial/ethnic subpopulations (e.g., Chinese, Vietnamese, Puerto Rican, or Mexican) to strengthen the design of prevention efforts and the provision of culturally and linguistically appropriate services.

## Conclusion

The recent increased attention on rural health issues presents an important opportunity for decreasing disparities in health and health care access between rural and urban communities. During 2016–2017, numerous organizations, including certain nonprofit organizations and federal agencies, focused more attention on rural health and urban-rural health disparities. For example, the Kaiser Family Foundation published numerous pieces on rural health, as did CDC (*30*) and another public health journal (*31*). In addition, the CMS Rural Health Council was established, as was the Interagency Task Force on Agriculture and Rural Prosperity, of which the U.S. Secretary of Health Human Services is a member.

The unique challenges to health and access to care experienced by rural racial/ethnic populations differ and are often overlooked when data are not analyzed for specific population groups. Missing this opportunity to identify and address underlying racial/ethnic disparities could lead to growing rural-urban disparities, even as new programs and partnerships seek to eliminate them. Conducting community health need assessments, implementing the national CLAS standards, and developing plans using the disparities action statement could improve outcomes for all rural residents. Such actions contribute to achieving health equity, which is one of the overarching *Healthy People 2020* goals.
